# Supplementary material for: Emergence and control of photonic band structure in stacked OLED microcavities
Source: Nat Commun. 2021 Oct 20;12:6111. doi: 10.1038/s41467-021-26440-3 (PMC8528838; doi:10.1038/s41467-021-26440-3)
Supplement: Supplementary file 4 — Supplementary Data 1 [file 41467_2021_26440_MOESM4_ESM.zip › OLED Simulation v2-1/OLED Simulation/Materials Data/Materials Database/rii-database-2019-02-11.zip!/database/info/main/SiO2.html]

# Silicon dioxide, SiO_2_

## Other names

- Quartz
- Silica
- Silicon oxide
- Silicon(IV) dioxide

## Polymorphs

- Alpha quartz (α-quartz, most common)
- Beta quartz (β-quartz, only stable at temperatures above 573 °C)
- Tridymite
- Cristobalite
- Coesite
- Stishovite
- Lechatelierite
- Chalcedony

## External links

- Silicon dioxide - Wikipedia
- Fused quartz - Wikipedia
- Silicon dioxide - WebElements
- Quartz - The Mineral and Gemstone Kingdom
